# The CsPHL11-CsPAL2 module mediates chitooligosaccharide-induced cold tolerance in cucumber seedlings

**DOI:** 10.3389/fpls.2026.1862603

**Published:** 2026-06-12

**Authors:** Wendi Meng, Luyao Zhang, Yidan Wang, Feng Li, Meilin Guo, Tao Liu, Zifan Zhao, Xueling Ye

**Affiliations:** College of Horticulture, Shenyang Agricultural University, Shenyang, China

**Keywords:** chitooligosaccharide, cold stress, cold tolerance, cucumber, phenylalanine ammonia-lyase

## Abstract

Cold stress is an important abiotic stressor that significantly affects cucumber production in protected cultivation. Although chitooligosaccharide (COS) is known to enhance plant stress tolerance, the precise molecular mechanisms underlying COS-mediated cold tolerance remain incompletely understood. In this study, we identified a novel transcriptional module, CsPHL11 (PHR1-LIKE 11)-CsPAL2, and elucidated its role in regulating cold response in cucumber seedlings. Exogenous application of COS robustly enhanced cold tolerance by suppressing the increases in relative electrolyte leakage and malondialdehyde levels, while promoting proline accumulation and antioxidant enzyme activities. Transcriptomic and physiological analyses revealed that COS acted as a potent elicitor, strongly inducing the expression of the MYB transcription factor *CsPHL11* under cold stress. CsPHL11 directly bound to the promoter of *CsPAL2* and activated its transcription, leading to elevated phenylalanine ammonia-lyase activity. This activation facilitated the accumulation of protective secondary metabolites, particularly total phenols and flavonoids, which efficiently scavenged excessive reactive oxygen species and preserved cell membrane integrity. Conversely, silencing of either *CsPHL11* or *CsPAL2* by virus-induced gene silencing significantly impaired cold tolerance and exacerbated oxidative damage. Overall, these results uncovered a previously unrecognized CsPHL11-CsPAL2 regulatory cascade, providing new mechanistic insights into COS-mediated cold tolerance and highlighting the potential of oligosaccharides for protecting cold-sensitive horticultural crops.

## 1 Introduction

Global climate fluctuations have precipitated frequent extreme cold weather events, establishing cold stress as a primary abiotic factor detrimental to the yield and quality of horticultural crops (Zeng et al., 2025). Cucumber (*Cucumis sativus* L.) is a globally significant vegetable crop that occupies a central role in protected cultivation across northern China (Di et al., 2025). As a typical thermophilic species, cucumber is highly susceptible to temperature fluctuations. Low temperatures inhibit photosynthesis in cucumber seedlings, a process accompanied by an excessive accumulation of reactive oxygen species (ROS) and elevated electrolyte leakage (Abe et al., 2012). Collectively, these adverse effects severely impair normal vegetative growth, development, and flower bud differentiation. Therefore, elucidating the molecular mechanisms underlying low-temperature tolerance in cucumber is imperative for improving both crop quality and yield.

Chitooligosaccharide (COS) is a chitosan oligomer typically characterized by a degree of polymerization below 20, generated through the hydrolysis of chitosan (Liaqat and Eltem, 2018). COS exhibits multiple bioactivities, including significant antioxidant (Li et al., 2013b), antibacterial (Li et al., 2013a), and antiviral properties (Kulikov et al., 2006). It is also recognized as an effective elicitor for promoting plant growth and enhancing tolerance to abiotic stresses (Zhou et al., 2018). Previous studies have demonstrated that COS can increase chlorophyll content and enhance photosynthetic performance in *Dendrobium orchid* (Limpanavech et al., 2008), promote mineral nutrient uptake in *Phaseolus vulgaris* (Chatelain et al., 2014), protect wheat from chilling injury by modulating antioxidant enzyme activities (Zou et al., 2017), and improve wheat growth under drought stress (Chen et al., 2024). However, it remains unclear how COS is involved in regulating stress tolerance in plants.

As the largest transcription factor (TF) family, MYB TFs act as important regulators in plant cold stress responses (Dubos et al., 2010). Several studies have successfully identified numerous MYB TFs in plants. *Arabidopsis* MYB14 and MYB15 are demonstrated as negative regulators of cold tolerance (Agarwal et al., 2006; Chen et al., 2013). MdMYB308L positively regulates cold tolerance by interacting with MdbHLH33 and enhancing its binding to the promoter of *MdCBF2* (*C-REPEAT/DRE BINDING FACTOR 2*) (An et al., 2020). DgMYB2 has been shown to directly target *DgGPX1* (*GLUTATHIONE PEROXIDASE 1*) to improve cold resistance in chrysanthemum (Yang et al., 2022). Furthermore, overexpression of *SpMYB1* gene enhanced cold stress tolerance in tomato (Jin et al., 2025). In rice, several MYB family members, including OsMYB2, OsMYB4, and OsMYB30, have been reported to be involved in cold stress regulation (Vannini et al., 2004; Yang et al., 2012; Lv et al., 2017; Mao et al., 2019).

Transcriptional reprogramming serves as a central mechanism in the plant response to cold stress. In cucumber, multiple regulatory pathways that modulate cold tolerance through transcriptional regulation have been identified. The suppression of either *CsGPA1* (*G PROTEIN ALPHA SUBUNIT 1*) or *CsCOR413PM2* (*COLD-REGULATED 413-PLASMA MEMBRANE 2*) decreased the expression of *CsICE* (*INDUCER OF CBF EXPRESSION*)-*CsCBF via* the *CsGPA1*-*CsCOR413PM2*-Ca²^+^ axis under low temperatures (Yan et al., 2022). Salicylic acid enhances cold tolerance in grafted cucumbers by promoting the expression of cold-responsive genes through the CsNPR1 (NONEXPRESSER OF PR GENES 1)-CsICE1 regulatory module (Fu et al., 2024). Additionally, the cold-activated transcription factor CsHHO2 (HRS1 HOMOLOG 2) directly binds to the promoter of *CsGR-RBP3* (GLYCINE-RICH RNA-BINDING PROTEIN 3) and activates its transcription to confer cold tolerance (Bin et al., 2024). Furthermore, CsEIN2 (ETHYLENE INSENSITIVE 2) activates *CsCBF2* expression through its interaction with CsEIN3 to enhance cold tolerance (Li et al., 2025a). Beyond transcriptional activation, epigenetic regulation also plays a pivotal role. CsRBOH5.1 (RESPIRATORY BURST OXIDASE HOMOLOG 5.1) facilitates H3K4me3 deposition and sustains cold-induced transcriptional memory following cold priming (Di et al., 2025).

Phenylalanine ammonia-lyase (PAL) is a rate-limiting regulatory enzyme that catalyzes the deamination of L-phenylalanine into trans-cinnamic acid, a critical initial step in plant secondary metabolism (MacDonald and D'Cunha, 2007). This reaction drives the phenylpropanoid pathway, which is established as one of the three fundamental secondary metabolic routes in plants. Consequently, PAL activity directly modulates the biosynthesis of downstream phenolic compounds, including lignin, flavonoids, isoflavones, and alkaloids. PAL genes significantly enhance plant tolerance and resistance under various abiotic and biotic stress conditions by regulating the synthesis of secondary metabolites and hormones (Dixon et al., 2002; Khakdan et al., 2018). In *Arabidopsis*, the *pal1 pal2 pal3 pal4* quadruple mutants exhibited reduced levels of salicylic acid and displayed increased susceptibility to *Pseudomonas syringae* (Huang et al., 2010). The overexpression of *OsPAL8* mediates resistance to the brown planthopper by regulating the biosynthesis and accumulation of salicylic acid and lignin (He et al., 2020). Similarly, the overexpression of *BcPAL1* and *BcPAL2* in non-heading Chinese cabbage significantly increased thermotolerance, accompanied by enhanced PAL activity (Gao et al., 2025). The *PAL* gene family in cucumber comprises 15 members divided into four clades (Gu et al., 2024), but the specific roles of the *CsPAL* gene family in the cucumber cold stress response remain largely unexplored.

Our previous studies indicated that the exogenous application of COS alleviates cold stress in cucumber seedlings, a process potentially mediated by the activation of the phenylpropanoid metabolic pathway (Tan et al., 2023). In the current study, we integrated multi-omics analyses, physiological assays, and molecular genetic approaches to elucidate the regulatory mechanisms by which COS enhances cold tolerance in cucumber seedlings. Our findings reveal a novel transcriptional module involving the MYB transcription factor CsPHL11 (PHR1-LIKE 11), which directly activates *CsPAL2* expression. Under cold stress, COS application strongly induced *CsPHL11* expression, leading to the transcriptional activation of *CsPAL2*. This cascade promotes PAL activity and the accumulation of protective metabolites, thereby enhancing cold tolerance. These findings uncover a previously unrecognized *CsPHL11*-*CsPAL2* transcriptional regulatory module that modulates PAL activity, providing new mechanistic insights into how COS confers cold tolerance in cucumber seedlings.

## 2 Materials and methods

### 2.1 Plant materials and growth conditions

Seeds of cucumber ‘Jinyan No. 4’ were soaked in 55 °C hot water for 10–15 min, then germinated in the dark at 28 °C. The germinated seeds were transferred to seedling trays placed in an incubator at 28 °C/18 °C (day/night) with a 12-h photoperiod and a photon flux density of 500 μmol m^-2^ s^-1^. At the first-true-leaf stage, robust seedlings were transplanted into nutrient bowls.

### 2.2 Chemical treatments and cold stress assays

To determine the optimal concentration of the PAL inhibitor for repressing cold tolerance, cucumber plants were pretreated with 1, 10, and 100 μM AIP (Aladdin, Shanghai, China), as well as 100 μM AOPP (Macklin, Shanghai, China). The plants were maintained at normal temperatures for 24 h and subjected to control or chilling stress conditions. The 100 μM AIP treatment, which resulted in the lowest cold tolerance, was subsequently used in the low-temperature experiments to elucidate the physiological mode of action. Exogenous substance applications were initiated when the seedlings developed their third true leaf. The experiment was performed in three independent biological replicates, with each treatment comprising 30 seedlings per replicate. Tween 20 (0.02% v/v; Sigma-Aldrich, St. Louis, MO, USA) was used as a surfactant during application. At the third-true-leaf stage, seedlings were pretreated with 50 mg·L^−^¹ exogenous COS (Sigma-Aldrich), 100 μM AIP, a combination of COS and AIP, or distilled water (control) at normal temperatures (28 °C/18 °C, day/night) for 24 h, after which they were exposed to cold stress (12 °C/6 °C, day/night). After 0, 6, 12, or 24 hours of cold stress, the fully expanded second and third leaves were selected for physiological and biochemical analysis.

### 2.3 Determination of physiological indicators

Physiological indicators of cold tolerance were determined as previously described (Tan et al., 2023). The relative electrolyte leakage (REC) was measured using an ORION TDS conductance meter (ORION Research, Inc., Franklin, MA, USA). The MDA and Pro contents were determined using the thiobarbituric acid colorimetric method and the acid ninhydrin method, respectively. The activities of POD and CAT were assessed using the guaiacol oxidation colorimetric method and standard colorimetric assays, respectively. Anthocyanin content was determined following established protocols (Fuleki and Francis, 1968). PAL activity was measured spectrophotometrically from plant extracts, as previously described (Shang et al., 2012). Total phenolics were extracted and determined as previously described (Meyers et al., 2003). A standard curve was generated using gallic acid, and the total phenol content in the samples was calculated accordingly. Flavonoid content was quantified using rutin as a standard (Zhishen et al., 1999).

### 2.4 qRT-PCR

Gene expression levels were analyzed by qRT-PCR using the SYBR qPCR Master Mix (Q713, Vazyme, Nanjing, China), and relative expression levels were calculated using the2^−△△Ct^ method (Zhao et al., 2025b). The cucumber *CsaV3_6G041900* gene served as the internal control (Liu et al., 2025). All gene sequences were obtained from the cucumber reference genome (http://www.cucumberdb.com/, Chinese Long v3.0). Three biological replicates and three technical replicates were performed for each sample.

### 2.5 Subcellular localization

The coding sequence (CDS) of *CsPAL2* was cloned into pCAMBIA1300-GFP vector, and the recombinant vector was introduced into *Nicotiana benthamiana* leaves by *Agrobacterium tumefaciens*-mediated infiltration. Transient transformation of tobacco leaves was performed as described previously (Zhao et al., 2025d). Fluorescence signals were observed using a confocal laser-scanning microscope (Leica TCS SP8, Wetzlar, Germany).

### 2.6 Virus-induced gene silencing assay

TRV-based VIGS was employed for functional verification in cucumber, following previously reported protocols (Bu et al., 2019) with an optimized injection method. Gene-specific fragments of *CsPAL2* and *CsPHL11* were cloned into the pTRV2 vector and transformed into *Agrobacterium tumefaciens* strain GV3101. The *A. tumefaciens* cultures were resuspended in an induction buffer containing 10 mM MgCl_2_, 10 mM MES, and 200 μM acetosyringone (OD600 = 1.0). The suspension carrying pTRV1 was mixed at a 1:1 ratio with that carrying pTRV2, pTRV2-CsPAL2, or pTRV2-CsPHL11. At the cotyledon expansion stage, the cotyledons were scratched with a blade and the mixed bacterial suspension was injected. The treated seedlings were then placed in the dark for 48 h. The silencing efficiencies of *CsPAL2* and *CsPHL11* were validated as previously reported (Zhao et al., 2025a).

### 2.7 Yeast one-hybrid assay

The *CsPAL2* promoter was cloned into the pAbAi vector, and the *CsPHL11* CDS was cloned into the pGADT7 vector. The *ProCsPAL2*-pAbAi construct was transformed into the yeast strain Y1HGold, and the minimal inhibitory concentration of Aureobasidin A (AbA) required for the *ProCsPAL2*-pAbAi bait strain was determined. Subsequently, the prey plasmid CsPHL11-pGADT7 was transformed into the bait strain. The experimental procedures followed previously described methods (Zhao et al., 2025c).

### 2.8 Dual-luciferase assay

The *CsPAL2* promoter was cloned into the pGreenII 0800-LUC vector (VT307, Coolaber) to generate the reporter construct, while the *CsPHL11* CDS was cloned into the pCAMBIA1300 vector to generate the effector construct. The dual-LUC assay was performed in accordance with previously described protocols (Zhao et al., 2026).

### 2.9 Statistical analysis

All quantitative data were analyzed using a one-way analysis of variance (ANOVA), and treatment means were compared using Tukey’s test. Different lowercase letters or asterisks indicate statistically significant differences at *P* < 0.05.

## 3 Results

### 3.1 Screening and concentration optimization of PAL inhibitors

Our previous studies demonstrated that the application of 50 mg·L^−^¹ COS significantly enhanced cold tolerance in cucumber seedlings. Transcriptome analysis revealed that differentially expressed genes (DEGs) induced by COS application under cold stress were significantly enriched in the phenylpropanoid biosynthesis pathway (ko00940) (Tan et al., 2023). PAL serves as the initial rate-limiting enzyme in this pathway, and extensive evidence suggests that PALs play essential roles in plant stress responses. Thus, we hypothesized that the COS-mediated enhancement of cold tolerance might be closely linked to PAL activity.

**Figure 1:**
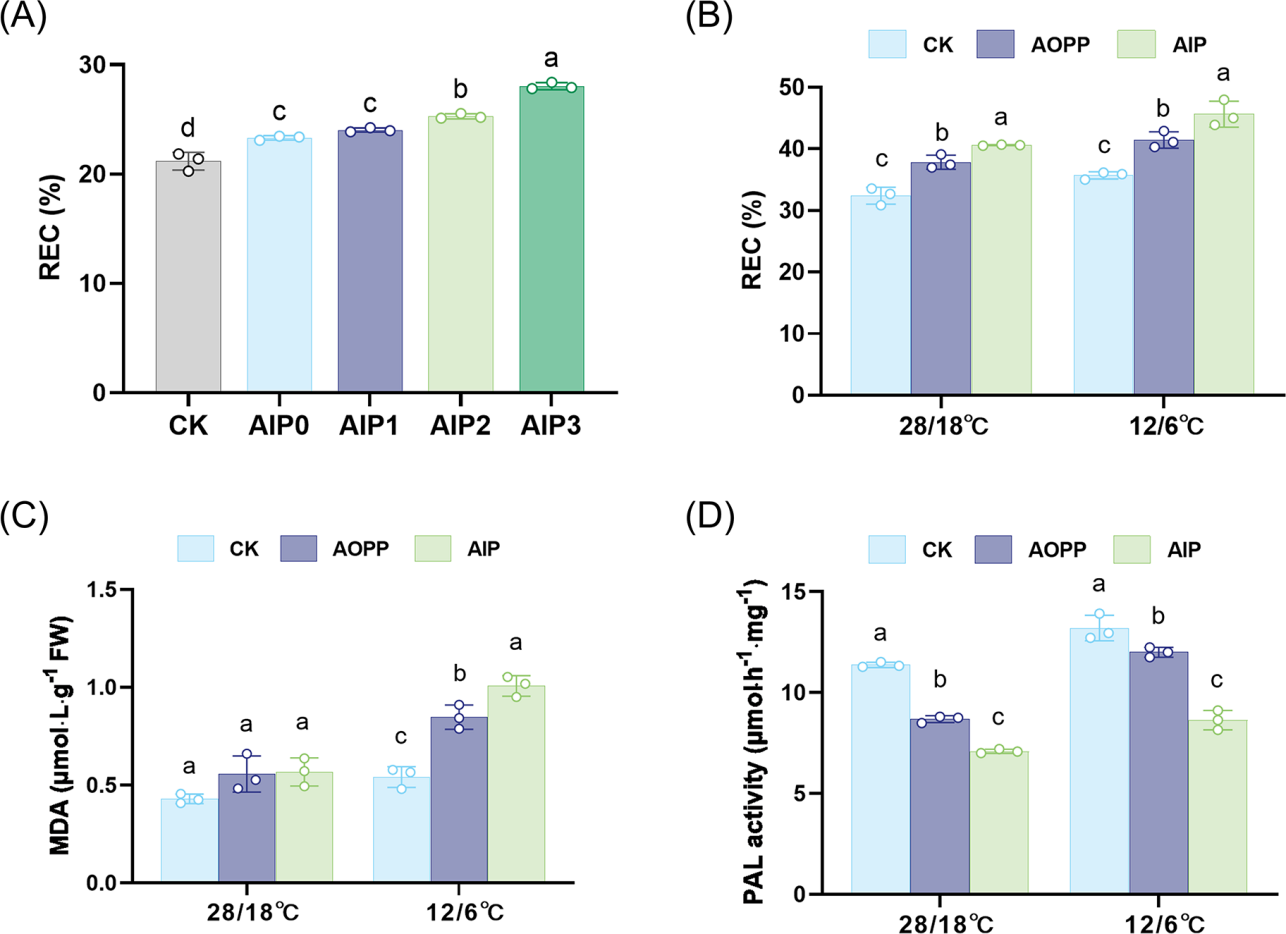
Effects of PAL inhibitors at different concentrations on cucumber seedlings. **(A)** Effects of different concentrations of AIP on cucumber seedlings CK: normal temperature control; AIP0, low temperature control without AIP; AIP1, 1 μM AIP under low temperature; AIP2, 10μM AIP under low temperature; AIP3, 100 μM AIP under low temperature. **(B)** Relative electrolyte leakage (REC), **(C)** malondialdehyde (MDA) content, and **(D)** phenylalanine ammonia-lyase (PAL) activity of cucumber seedlings under normal temperature and low temperature. Data are presented as means ± SD (n = 3). Different lowercase letters Indicate statistically significant differences (P < 0.05) within the groups as determined by one-way ANOVA followed by Duncan's multiple range tests. CK, control; COS, chitooligosaccharide; AIP, 2-aminoindan-2-phosphonic acid.

To test this hypothesis, we first compared the impact of different PAL inhibitors on cucumber under cold stress. 2-aminoindan-2-phosphonic acid (AIP) is a well-characterized competitive PAL inhibitor (Jones and Saxena, 2013). To determine the optimal concentration of AIP for subsequent cold stress experiments, cucumber seedlings were treated with a range of AIP concentrations (1, 10, and 100 μM). Under cold stress, REC was lowest in the control group (CK; 0 μM AIP) and gradually increased with rising AIP concentration, peaking in the 100 μM treatment (**Figure 1A**). Accordingly, 100 μM AIP was selected as the optimal concentration for inhibiting cold tolerance in subsequent assays. AOPP is also recognized as a PAL inhibitor (Pérez-Pérez et al., 2025). To compare the effects of AOPP and AIP on cold tolerance in cucumber seedlings, we further analyzed the REC, MDA content, and PAL activity under low-temperature conditions. The results demonstrated that, compared with the control, both inhibitors significantly elevated REC and MDA levels and inhibited PAL activity (**Figures 1B–D**). Moreover, compared with AOPP treatment, AIP treatment resulted in a stronger inhibitory effect on the PAL activity and cold tolerance of cucumber seedlings. Thus, 100 μM AIP was selected for use in subsequent experiments.

### 3.2 PAL activity is required for cold tolerance in cucumber seedlings

**Figure 2:**
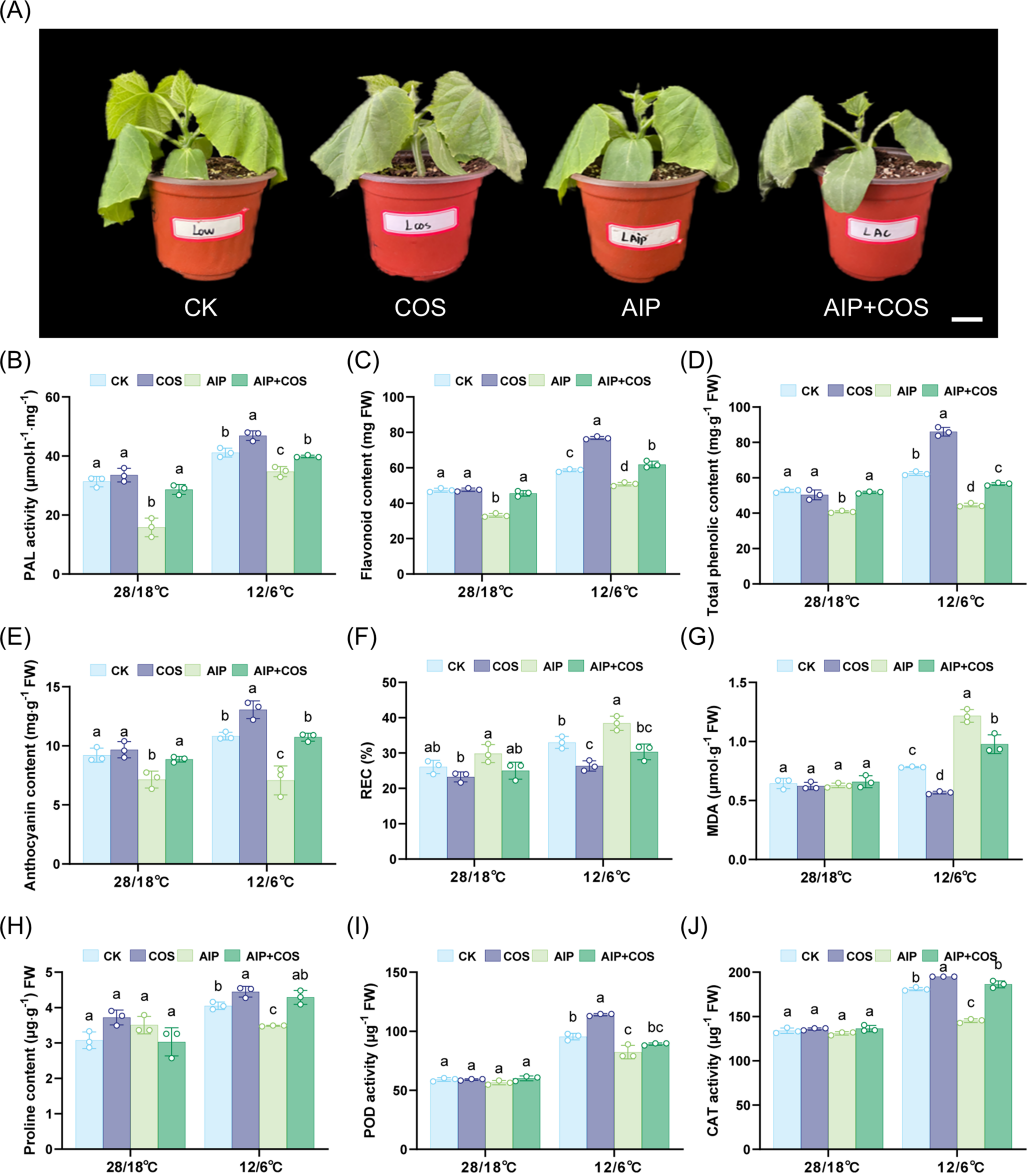
Effects of exogenous COS and PAL inhibitor on cold tolerance of cucumber seedlings under cold stress. **(A)** Phenotypic comparison of cucumber seedlings subjected to different treatments (CK, COS, AIP, COS+AIP) under normal and low temperature conditions. Bars=2 cm. **(B)** phenylalanine ammonia-lyase (PAL) activity, **(C)** flavonoid content, **(D)** total phenolic content, **(E)** anthocyanin content, **(F)** relative electrolyte leakage (REC), **(G)** malondialdehyde (MDA) content, **(H)** Proline **(I)** POD activity, and **(J)** CAT activity in cucumber seedlings subjected to different treatments under normal and cold stress conditions. Data are presented as means ± SD (n = 3). Different lowercase letters indicate statistically significant differences (*P* < 0.05) within the groups as determined by one-way ANOVA followed by Duncan’s multiple range tests. CK, control; COS, chitooligosaccharide; AIP, 2-aminoindan-2-phosphonic acid; AIP+COS, a combination of AIP and COS.

We then subjected the seedlings to COS, AIP, and a combination of AIP and COS (AIP+COS) treatments under both ambient and low-temperature conditions. Compared with the control group, the COS treatment significantly enhanced the cold tolerance of the seedlings, while the AIP treatment impaired it (**Figure 2A**). Furthermore, the application of COS partially mitigated the inhibitory effect of AIP on the cold tolerance of cucumber seedlings. Concurrently, under low-temperature conditions, the COS treatment increased PAL activity and the accumulation of phenylpropanoid metabolites (such as total phenols and flavonoids) in cucumber seedlings (**Figures 2B–E**). Furthermore, COS treatment reduced REC and MDA content, while increasing Pro levels and antioxidant enzyme activities (**Figures 2F–I**). Conversely, AIP had the opposite effects, which were partially reversed by the combined AIP+COS treatment (**Figures 2F–I**). These results indicated that PAL activity is positively correlated with the cold tolerance of cucumber seedlings and that this activity is effectively induced by exogenous COS.

### 3.3 Exogenous COS treatment upregulates the expression of *CsPAL2*

**Figure 3:**
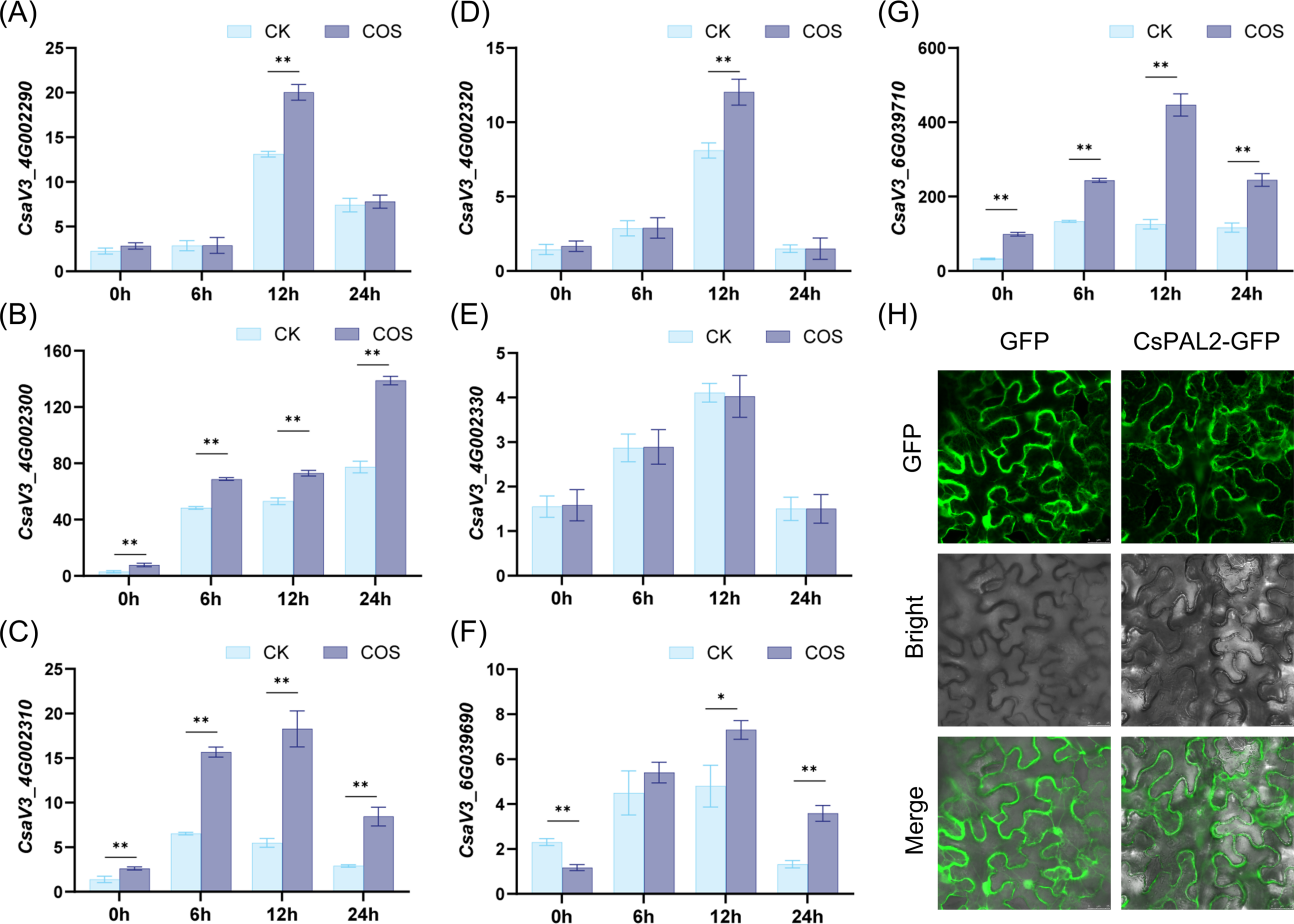
Exogenous chitosan chitooligosaccharide upregulates the expression of *CsPAL2* under cold stress. **(A–G)** The expression levels of PAL genes in cucumber seedlings under low temperature conditions. Data are presented as means ± SD (n = 3). Asterisks indicate significant differences compared with the control (2-tailed Student’s *t*-test, *P < 0.05, **P < 0.01). CK, treated without chitooligosaccharide; COS, treated with chitooligosaccharide. **(H)** Subcellular localization of CsPAL2 protein. 35S::GFP was used as control.

To elucidate the molecular mechanism underlying COS-mediated cold tolerance in cucumber, we first focused on PAL genes and found that 7 genes encoding PAL proteins were markedly upregulated following COS treatment. To explore the role of these PAL proteins in cold stress response, we examined the transcript levels of the PAL genes in cucumber grown under normal conditions (28 °C/18 °C) or exposed to cold stress (12 °C/6 °C) for 6 h, 12h, and 24 h (**Figures 3A–G**). Among them, the *CsaV3_6G039710* (*CsPAL2*) gene exhibited the most robust and sustained upregulation in response to COS treatment under cold stress. In addition, the CsPAL2-GFP fusion protein driven by the CaMV 35S promoter was transiently expressed in tobacco leaves. The protein was localized to the cytoplasm (**Figure 3H**), consistent with its role. Therefore, *CsPAL2* was prioritized for further functional characterization.

### 3.4 Silencing of *CsPAL2* impairs cold tolerance in cucumber seedlings

**Figure 4:**
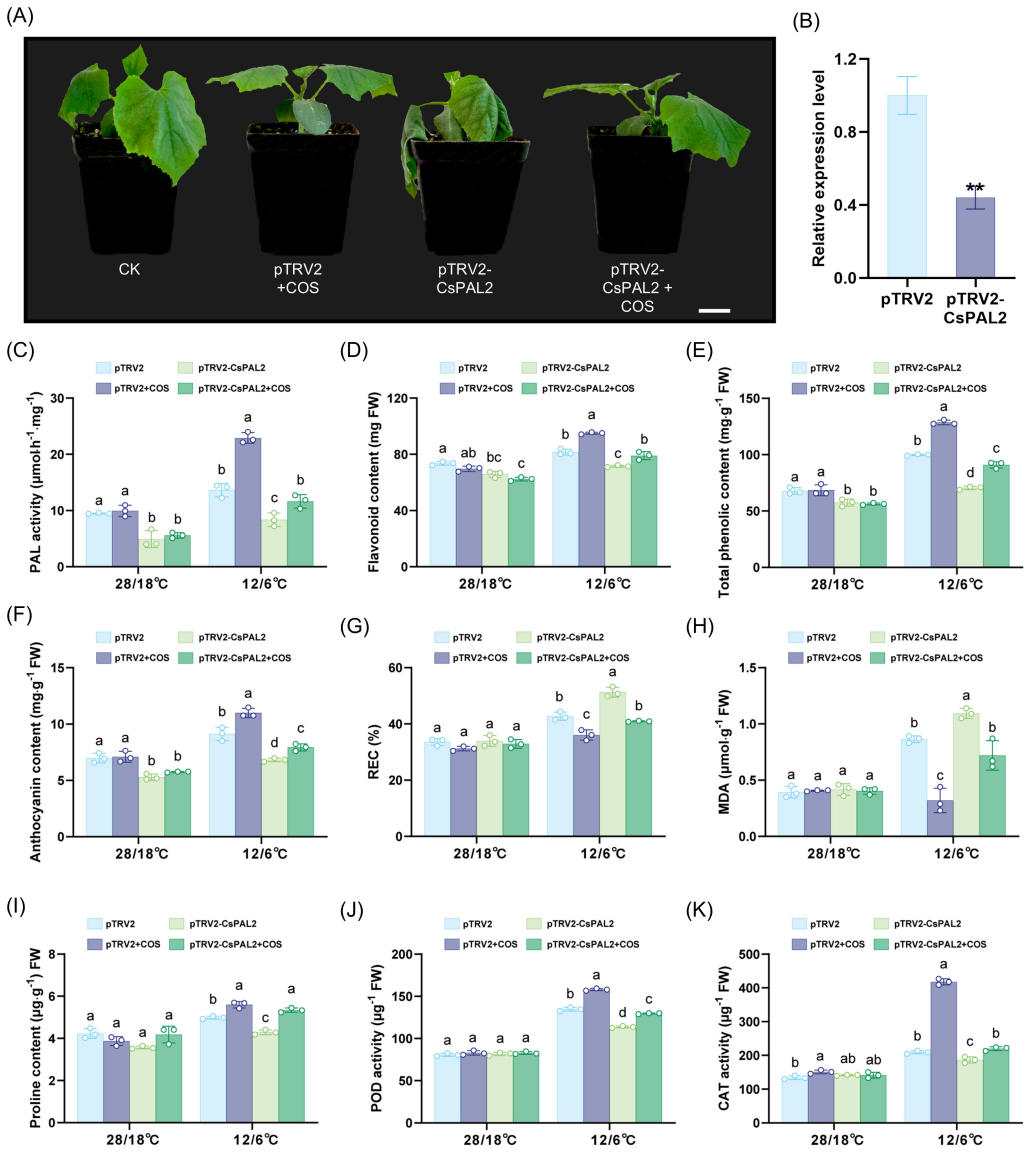
*CsPAL2* positively regulates cold stress responses in cucumber seedlings. **(A)** Phenotypic comparison of pTRV2 control and pTRV2-CsPAL2 lines under low temperature conditions. Bars=2 cm. **(B)** The relative expression level of CsPAL2 in pTRV2 and pTRV2-CsPAL2 seedlings. Data are presented as means ± SD (n = 3). Asterisks indicate significant differences compared with the control (2-tailed Student’s *t*-test, **P < 0.01). **(C)** phenylalanine ammonia-lyase (PAL) activity, **(D)** flavonoid content, **(E)** total phenolic content, **(F)** anthocyanin content, **(G)** relative electrolyte leakage (REC), **(H)** malondialdehyde (MDA) content, **(I)** Proline content, **(J)** POD activity, and **(K)** CAT activity in pTRV2 and pTRV2-CsPAL2 cucumber seedlings subjected to different treatments under normal and cold stress conditions. Data are presented as means ± SD (n = 3). Different lowercase letters indicate statistically significant differences (*P* < 0.05) within the groups as determined by one-way ANOVA followed by Duncan’s multiple range tests.

To functionally characterize *CsPAL2*, we silenced its expression in cucumber seedlings and exposed the plants to cold stress to verify the hypothesis that *CsPAL2* contributes to chilling tolerance (**Figure 4A**). The transcript level of *CsPAL2* in the silenced line was reduced to 45% of that observed in the empty vector control plants (**Figure 4B**). Phenotypic analysis indicates that the silencing of *CsPAL2* significantly exacerbated the wilting caused by cold stress (**Figure 4A**). Under low-temperature conditions, the silenced plants accumulated fewer metabolites involved in the phenylpropanoid biosynthesis pathway and exhibited reduced PAL activity (**Figures 4C-E**). Consistently, *CsPAL2*-silenced plants displayed higher REC and MDA content, and lower Pro accumulation and antioxidant enzyme activities following chilling exposure (**Figure 4**). Crucially, the compensatory effect of exogenous COS on these physiological parameters was substantially impaired in the CsPAL2-silenced seedlings. Collectively, these results demonstrated that *CsPAL2* plays a positive regulatory role in cold tolerance, and that exogenous COS can mediate the activation of the phenylpropanoid metabolic pathway through CsPAL2, thereby alleviating the damage caused by cold stress.

### 3.5 Identification of *CsPHL11* as an upstream regulator of *CsPAL2* in response to COS

**Figure 5:**
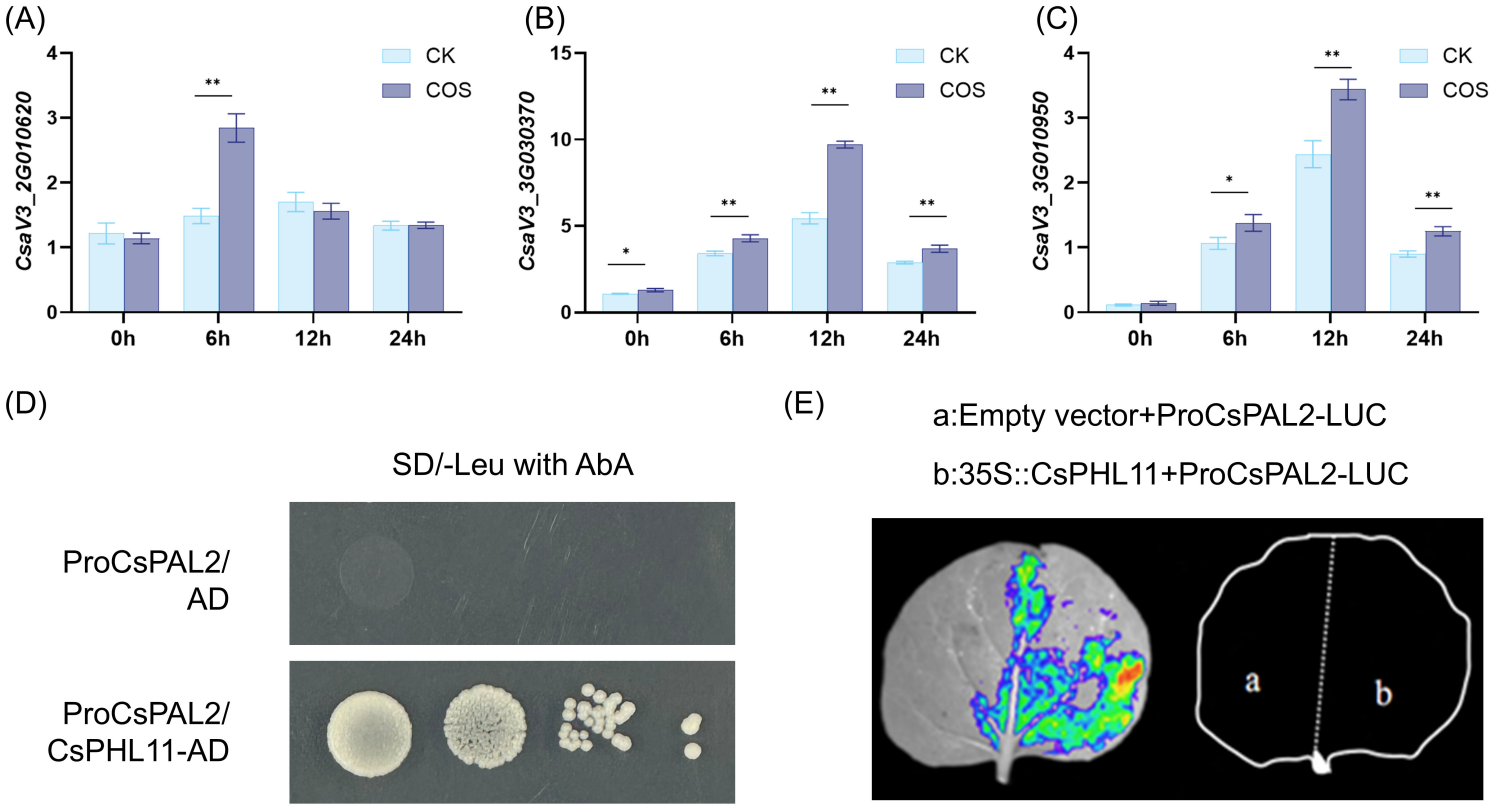
CsPHL11 responds to chitooligosaccharide under low temperature and activates *CsPAL2* expression by directly binding to its promoter. Relative expression levels of **(A)** *CsPHL8* **(B)** *CsPHL11*, and **(C)** *CsPHL2* in cucumber seedlings under normal and cold stress conditions. Data are presented as means ± SD (n = 3). Asterisks indicate significant differences compared with the control (2-tailed Student’s *t*-test, *P < 0.05, **P < 0.01). **(D)** Yeast one-hybrid assay demonstrating that CsPHL11 binds to the *CsPAL2* promoter. **(E)** Dual-luciferase assays showing activation of the *CsPAL2* promoter by CsPHL11.

To elucidate the regulatory mechanism by which exogenous COS enhances cold tolerance in cucumber seedlings, we conducted an analysis of the *CsPAL2* promoter region using the PlantCARE database. This analysis revealed the presence of multiple putative binding sites for MYB transcription factors. Given the established roles of MYB family members in cold stress responses, we screened our transcriptome data and identified three MYB transcription factor genes, *CsaV3_2G010620* (*CsPHL8*), *CsaV3_3G030370* (*CsPHL11*), and *CsaV3_3G010950* (*CsPHL2*), whose expression patterns closely paralleled that of *CsPAL2* under COS treatment (**Figures 5A-C**). To validate this co-expression pattern, we examined the transcript levels of these genes in response to exogenous COS using qRT-PCR. Our qRT-PCR results confirmed that only *CsPHL2* and *CsPHL11* exhibited expression dynamics similar to that of *CsPAL2*, with transcript levels increasing transiently upon COS treatment and subsequently declining during prolonged cold exposure. Notably, *CsPHL11* showed a stronger and more consistent response to COS treatment than *CsPHL2*. Based on this pronounced co-expression pattern, we hypothesized that *CsPHL11* may act as a potential upstream regulator of *CsPAL2*.

### 3.6 CsPHL11 directly activates the transcription of *CsPAL2*

Given that the expression pattern of *CsPHL11* was highly synchronized with that of *CsPAL2* following COS treatment under low temperature (Tan et al., 2023), we hypothesized that CsPHL11 acts upstream of *CsPAL2*. To test this, the CDS of *CsPHL11* was cloned into the pGADT7 vector to generate the CsPHL11-AD construct, and a Y1H assay was performed to examine whether CsPHL11 binds to the *CsPAL2* promoter. Yeast cells co-transformed with CsPHL11-AD and the *ProCsPAL2* reporter grew well on selective medium, indicating that CsPHL11 directly binds to the *CsPAL2* promoter (**Figure 5D**). Furthermore, a dual-LUC assay was conducted in *Nicotiana benthamiana* leaves to investigate the regulatory effect of CsPHL11 on *CsPAL2* transcription. Co-infiltration of 35S::CsPHL11 with *ProCsPAL2::LUC* resulted in a significant increase in luminescence compared with the control, suggesting that CsPHL11 functions as a transcriptional activator of *CsPAL2* (**Figure 5E**). Taken together, these results demonstrated that CsPHL11 directly binds to the *CsPAL2* promoter and activates its transcription.

### 3.7 Silencing of *CsPHL11* reduces cold tolerance in cucumber

**Figure 6:**
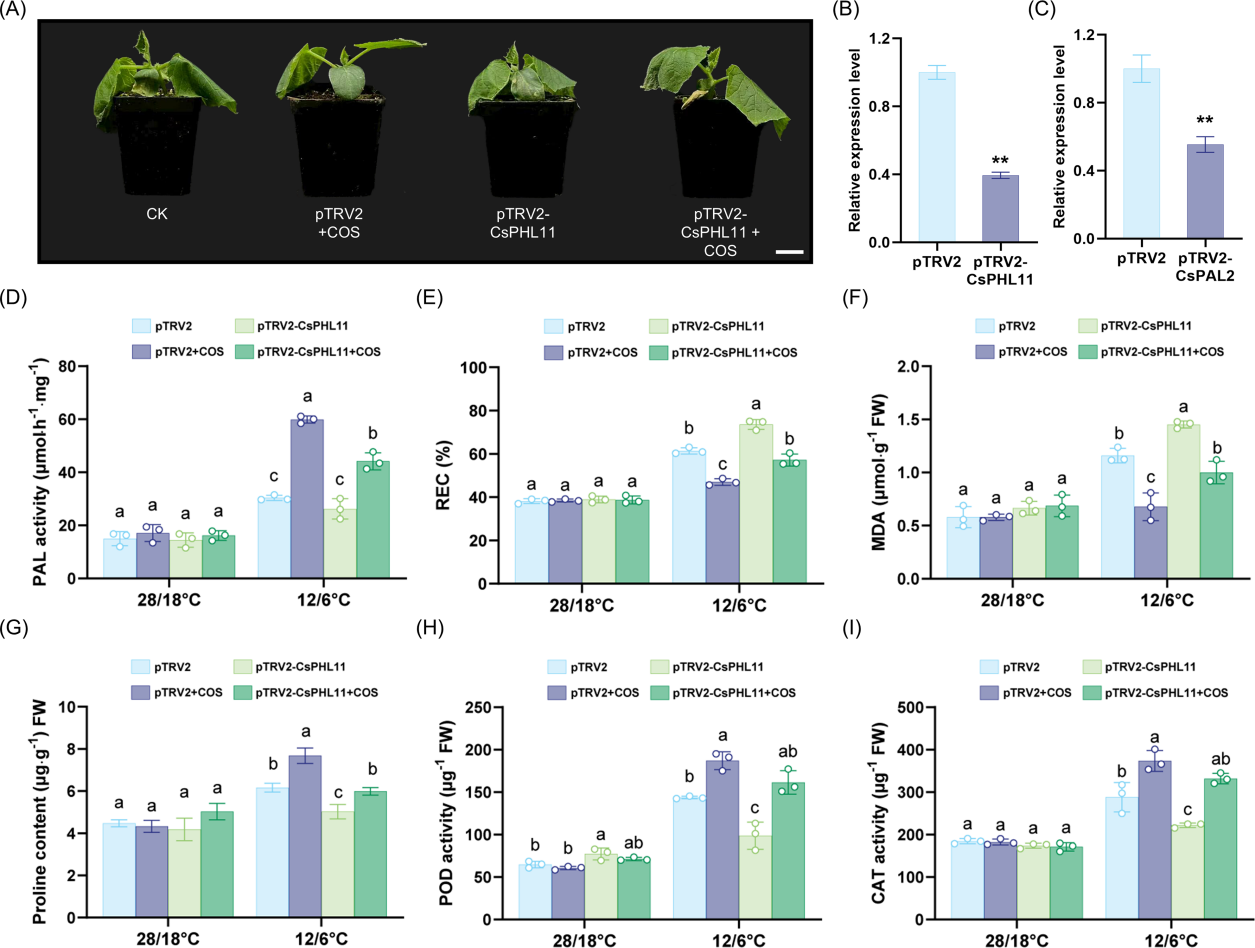
*CsPHL11* positively regulates cold stress responses in cucumber seedlings. **(A)** Phenotypic comparison of pTRV2 control and pTRV2-CsPHL11 lines under low temperature conditions. Bars=2 cm. The relative expression level of **(B)** *CsPHL11* and **(C)** *CsPAL2* in pTRV2 and pTRV2-CsPHL11 seedlings. Data are presented as means ± SD (n = 3). Asterisks indicate significant differences compared with the control (2-tailed Student’s *t*-test, **P < 0.01). **(D)** phenylalanine ammonia-lyase (PAL) activity, **(E)** relative electrolyte leakage (REC), **(F)** malondialdehyde (MDA) content, **(G)** Proline **(H)** POD activity, and **(I)** CAT activity in pTRV2 and pTRV2-CsPHL11 cucumber seedlings subjected to different treatments under normal and cold stress conditions. Data are presented as means ± SD (n = 3). Different lowercase letters indicate statistically significant differences (*P* < 0.05) within the groups as determined by one-way ANOVA followed by Duncan’s multiple range tests.

To investigate the biological functions of *CsPHL11* in cucumber cold tolerance, we silenced *CsPHL11* in cucumber seedlings using VIGS (**Figure 6A**). The qRT-PCR results confirmed that *CsPHL11* transcript levels were significantly reduced in the pTRV2-CsPHL11 plants compared to the pTRV2 empty-vector control (**Figure 6B**). Phenotypically, compared with the control plants, the *CsPHL11*-silenced plants exhibited severe wilting following cold treatment. Consistent with the significant downregulation of *CsPAL2* expression, the pTRV2-CsPHL11 plants exhibited lower CsPAL2 expression levels and diminished PAL activity (**Figures 6C, D**). Furthermore, the *CsPHL11*-silenced plants exhibited higher REC and MDA levels, alongside lower Pro contents and antioxidant enzyme activities compared to the pTRV2 plants (**Figures 6F-I**). Collectively, these results demonstrate that *CsPHL11* positively regulates cold tolerance in cucumber seedlings by directly activating *CsPAL2* expression.

## 4 Discussion

Cold stress is a primary environmental factor that severely limits the growth, development, and geographical distribution of cucumber (Yan et al., 2022). Therefore, developing cold-tolerant varieties through genetic engineering or identifying effective exogenous protectants is crucial for the protected cultivation of this crop. Accumulating evidence suggests that application of exogenous substances is a key strategy for reducing low-temperature damage in cultivated plants. COS has been demonstrated to be an effective inducer that promotes plant growth and enhances tolerance to abiotic stress (Zhou et al., 2018). Despite previous studies showing that COS could enhance cold tolerance in plant species (Zhang et al., 2019; Li et al., 2020), the molecular mechanisms are still unclear. Elucidating the regulatory modules is therefore essential to understand how COS application confers cold tolerance in cucumber seedlings. To address this gap, our study investigated the regulatory networks activated by exogenous COS application under low-temperature conditions. Our results demonstrate the essential role of the COS-induced CsPHL11-CsPAL2 module in enhancing cold tolerance in cucumber seedlings.

Through transcriptomic analysis, we found that the application of COS under low-temperature conditions led to an accumulation of *CsPAL2* transcripts. We identified CsPAL2 as a positive regulator in cold tolerance, and the function of *CsPAL2* was confirmed by VIGS assays. PAL is the first rate-limiting enzyme that catalyzes the deamination of L-phenylalanine into trans-cinnamic acid in thephenylpropanoid biosynthesis pathway in plants (MacDonald and D'Cunha, 2007). The exogenous COS-induced PAL activity enhances stress tolerance in cucumber, consistent with findings under salt and drought stresses (Fan et al., 2022; Qin et al., 2022). In contrast, the lower PAL activity in pTRV2-CsPAL2 plants reduced cucumber cold tolerance and compromised the protective effects of exogenous COS. Furthermore, exogenous COS still partially enhanced the cold tolerance in *CsPAL2*-silenced plants, suggesting that COS may also activate PAL2-independent signaling pathways to improve cold tolerance. However, further investigations are required to elucidate these comprehensive signaling networks.

Notably, an MYB binding site was identified in the *CsPAL2* promoter, suggesting that an MYB TF might directly mediate the COS signal. Expression patterns confirmed that *CsPHL11*, an MYB TF, was strongly induced by COS and exhibited a similar pattern to that of *CsPAL2*. Y1H and dual-LUC assays further demonstrated that CsPHL11 directly bound to the *CsPAL2* promoter and activated its expression. MYB TFs regulate the phenylalanine metabolic pathway by participating in the biosynthesis of anthocyanins, flavonoids, and lignin, and are essential for various developmental pathways and stress responses (Pratyusha and Sarada, 2022). These results suggest that CsPHL11 may upregulate *CsPAL2* expression through a COS-dependent pathway, thereby promoting cold tolerance in cucumber seedlings.

Phenylpropanoid metabolites, including phenolics and flavonoids, effectively scavenge ROS and stabilize cellular structures under abiotic stress. Low temperatures activate the phenylpropanoid-flavonoid metabolic pathway, leading to increased anthocyanin content, which mitigates oxidative damage and prevents leaf yellowing (Petridis et al., 2016; Lu et al., 2019; Naing and Kim, 2021; Pratyusha and Sarada, 2022). Similarly, cold stress upregulates the expression of flavonoid metabolism-related genes in *Tetrastigma hemsleyanum*, increasing total flavonoid content and antioxidant capacity (Peng et al., 2019). Our results indicate that the regulation of the upstream phenylalanine metabolism pathway is a key factor in cold tolerance of cucumber seedlings. Both AIP-mediated inhibition of PAL activity and silencing of *CsPAL2* reduced cold tolerance and caused cellular damage. In contrast, the high expression of *CsPAL2* induced by exogenous COS promotes the accumulation of phenolic compounds and flavonoids, thereby enhancing cold tolerance.

Cold stress damages cell membranes, leading to osmotic imbalance and lipid peroxidation, which manifest as wilting, increased REC, and MDA accumulation (Tan et al., 2026). It also triggers a massive accumulation of reactive oxygen species (ROS) and induces oxidative stress (Naing and Kim, 2021). Plants initiate a series of defense responses, including the accumulation of osmoregulatory substances such as proline and the induction of protective secondary metabolites to counteract this damage (Zhang et al., 2014; Jiao et al., 2021; Xing et al., 2025). In many horticultural crops, the activation of specific secondary metabolic pathways or the accumulation of signaling molecules can increase the activity of antioxidant enzymes such as SOD and POD, thereby alleviating oxidative damage (Chen et al., 2008; Quan et al., 2022; Ding et al., 2024; Li et al., 2025b; Zhang et al., 2026). We found that exogenous COS treatment suppressed the abnormal increases in REC and MDA induced by low temperatures, while simultaneously increasing proline content and antioxidant enzyme activity. Conversely, silencing *CsPAL2* or its upstream transcription factor *CsPHL11* could eliminate these protective responses and exacerbate cellular damage under cold stress. COS mitigates cold-induced damage by stabilizing cell membranes. Therefore, we propose that exogenous COS promotes the synthesis of phenylpropanoid metabolites *via* the CsPHL11-CsPAL2 module. These metabolites contribute to the improvement of the antioxidant defense system of cucumber.

**Figure 7:**
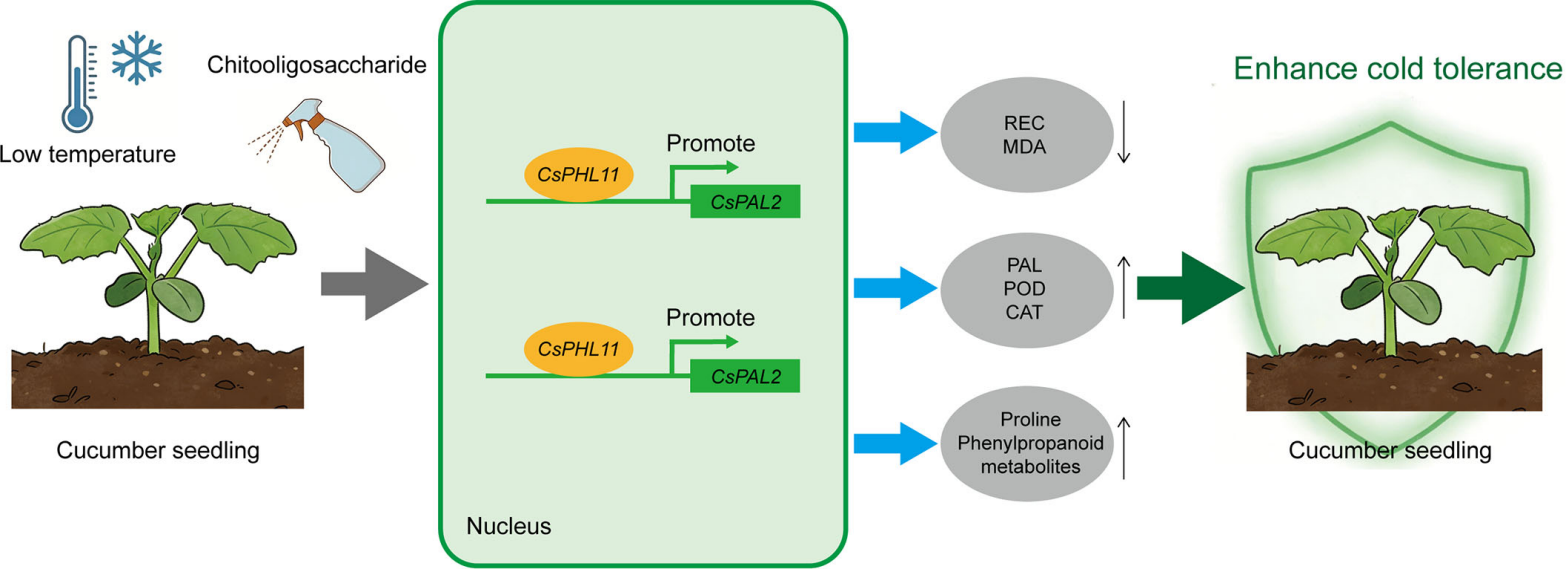
A working model of the COS-induced CsPHL11-CsPAL2 regulatory module enhancing cold tolerance in cucumber seedlings. Under low-temperature conditions, exogenous COS induces the expression of the MYB transcription factor *CsPHL11*. CsPHL11 directly binds to the *CsPAL2* promoter and activates its transcription, increasing PAL enzyme activity and promoting the synthesis of phenylpropanoid metabolites such as phenols and flavonoids. These secondary metabolites scavenge ROS and protect cell membrane integrity, thereby enhancing cold tolerance in cucumber seedlings.

In summary, this study proposes a schematic model describing the COS-induced CsPHL11-CsPAL2 regulatory module that governs cold tolerance in cucumber seedlings (**Figure 7**). Under low-temperature conditions, exogenous COS acts as a signaling molecule to strongly activate the expression of the MYB transcription factor *CsPHL11*. CsPHL11 subsequently directly binds to and activates *CsPAL2*, increasing PAL activity and accelerating the synthesis of phenylpropanoids. These compounds enhance the scavenging of reactive oxygen species and maintain cell membrane stability. This study reveals a transcriptional regulatory network linking COS signaling to phenylpropanoid metabolism, highlighting the potential for using oligosaccharides to enhance cold tolerance in cold-sensitive crops.

## Data Availability

The original contributions presented in the study are included in the article/**Supplementary Material**. Further inquiries can be directed to the corresponding author.

## References

[B1] Abe A. Kosugi S. Yoshida K. Natsume S. Takagi H. Kanzaki H. . (2012). Genome sequencing reveals agronomically important loci in rice using MutMap. Nat. Biotechnol. 30, 174–178. doi: 10.1038/nbt.2095 22267009

[B2] Agarwal M. Hao Y. Kapoor A. Dong C. H. Fujii H. Zheng X. . (2006). A R2R3 type MYB transcription factor is involved in the cold regulation of CBF genes and in acquired freezing tolerance. J. Biol. Chem. 281, 37636–37645. doi: 10.1074/jbc.M605895200 17015446

[B3] An J. Wang X. Zhang X. Xu H. Bi S. You C. . (2020). An apple MYB transcription factor regulates cold tolerance and anthocyanin accumulation and undergoes MIEL1-mediated degradation. Plant Biotechnol. J. 18, 337–353. doi: 10.1111/pbi.13201 31250952 PMC6953192

[B4] Bin W. Wang G. Wang Y. Jiang Y. Zhu Y. He J. . (2024). A cold-inducible MYB like transcription factor, CsHHO2, positively regulates chilling tolerance of cucumber fruit by enhancing CsGR-RBP3 expression. Postharvest Biol. Technol. 218, 113172. doi: 10.1016/j.postharvbio.2024.113172 38826717

[B5] Bu R. Wang R. Wei Q. Hu H. Sun H. Song P. . (2019). Silencing of glycerol-3-phosphate acyltransferase 6 (GPAT6) gene using a newly established virus induced gene silencing (VIGS) system in cucumber alleviates autotoxicity mimicked by cinnamic acid (CA). Plant Soil 438, 329–346. doi: 10.1007/s11104-019-03996-0

[B6] Chatelain P. G. Pintado M. E. Vasconcelos M. W. (2014). Evaluation of chitooligosaccharide application on mineral accumulation and plant growth in Phaseolus vulgaris. Plant Sci. 215-216, 134–140. doi: 10.1016/j.plantsci.2013.11.009 24388524

[B7] Chen G. Shangguan W. Chen H. Xu C. Bilal M. Zhao P. . (2024). Chitooligosaccharide modified pesticide-loaded polyurethane microcapsules to mitigate drought stress in wheat. Chem. Eng. J. 479, 147688. doi: 10.1016/j.cej.2023.147688 38826717

[B8] Chen T. F. Zheng W. J. Wong Y. S. Yang F. (2008). Selenium-induced changes in activities of antioxidant enzymes and content of photosynthetic pigments in Spirulina platensis. J. Integr. Plant Biol. 50, 40–48. doi: 10.1111/j.1744-7909.2007.00600.x 18666950

[B9] Chen Y. Chen Z. Kang J. Kang D. Gu H. Qin G. (2013). AtMYB14 regulates cold tolerance in Arabidopsis. Plant Mol. Biol. Rep. 31, 87–97. doi: 10.1007/s11105-012-0481-z 24415840 PMC3881570

[B10] Di Q. Zhou M. Li Y. Yan Y. He C. Wang J. . (2025). RESPIRATORY BURST OXIDASE HOMOLOG 5.1 regulates H3K4me3 deposition and transcription after cold priming in cucumber. Plant Physiol. 197 (2), kiae461. doi: 10.1093/plphys/kiae461 39208445

[B11] Ding X. Ma J. Liu S. Dong X. Pan X. Dong B. (2024). Acid electrolytic water treatment improves the quality of fresh-cut red pitaya fruit by regulating ROS metabolism and phenylpropanoid pathway. Postharvest Biol. Technol. 207, 112636. doi: 10.1016/j.postharvbio.2023.112636 38826717

[B12] Dixon R. A. Achnine L. Kota P. Liu C. J. Reddy M. S. Wang L. (2002). The phenylpropanoid pathway and plant defence-a genomics perspective. Mol. Plant Pathol. 3, 371–390. doi: 10.1046/j.1364-3703.2002.00131.x 20569344

[B13] Dubos C. Stracke R. Grotewold E. Weisshaar B. Martin C. Lepiniec L. (2010). MYB transcription factors in Arabidopsis. Trends Plant Sci. 15, 573–581. doi: 10.1016/j.tplants.2010.06.005 20674465

[B14] Fan L. Shi G. Yang J. Liu G. Niu Z. Ye W. . (2022). A Protective Role of Phenylalanine Ammonia-Lyase from Astragalus membranaceus against Saline-Alkali Stress. Int. J. Mol. Sci. 23 (24), 15686. doi: 10.3390/ijms232415686 36555329 PMC9779599

[B15] Fu X. Feng Y. Zhang Y. Bi H. Ai X. (2024). Salicylic acid improves chilling tolerance via CsNPR1-CsICE1 interaction in grafted cucumbers. Hortic. Res. 11, uhae231. doi: 10.1093/hr/uhae231 39434831 PMC11492142

[B16] Fuleki T. Francis F. J. (1968). Quantitative methods for anthocyanins. J. Food Sci. 33, 72–77. doi: 10.1111/j.1365-2621.1968.tb00887.x 40046247

[B17] Gao Z. Wang H. Chen X. Ding Q. Li E. Shen Y. . (2025). BcVQ11A-BcWRKY23-BcWRKY25 module is involved in thermotolerance by regulating phenylalanine ammonia-lyase activity in non-heading Chinese cabbage. Plant Cell Environ. 48, 2357–2376. doi: 10.1111/pce.15301 39601112

[B18] Gu J. Sohail H. Qiu L. Chen C. Yue H. Li Z. . (2024). Genome-wide characterization and expression analysis of CsPALs in cucumber (Cucumis sativus L.) reveal their potential roles in abiotic stress and aphid stress tolerance. Plants (Basel) 13 (18), 2537. doi: 10.3390/plants13182537 39339512 PMC11435200

[B19] He J. Liu Y. Yuan D. Duan M. Liu Y. Shen Z. . (2020). An R2R3 MYB transcription factor confers brown planthopper resistance by regulating the phenylalanine ammonia-lyase pathway in rice. Proc. Natl. Acad. Sci. U.S.A. 117, 271–277. doi: 10.1073/pnas.1902771116 31848246 PMC6955232

[B20] Huang J. Gu M. Lai Z. Fan B. Shi K. Zhou Y.-H. . (2010). Functional analysis of the Arabidopsis PAL gene family in plant growth, development, and response to environmental stress. Plant Physiol. 153, 1526–1538. doi: 10.1104/pp.110.157370 20566705 PMC2923909

[B21] Jiao C. Lan G. Sun Y. Wang G. Sun Y. (2021). Dopamine alleviates chilling stress in watermelon seedlings via modulation of proline content, antioxidant enzyme activity, and polyamine metabolism. J. Plant Growth Regul. 40, 277–292. doi: 10.1007/s00344-020-10096-2 30311153

[B22] Jin R. Muhammad T. Jia C. Yang T. Yang H. Wang J. . (2025). Overexpression of R2R3-MYB type transcription factor SpMYB1 enhances cold and drought tolerance in tomato. Plant Physiol. Biochem. 229, 110326. doi: 10.1016/j.plaphy.2025.110326 40782576

[B23] Jones A. M. Saxena P. K. (2013). Inhibition of phenylpropanoid biosynthesis in Artemisia annua L.: a novel approach to reduce oxidative browning in plant tissue culture. PloS One 8, e76802. doi: 10.1371/journal.pone.0076802 24116165 PMC3792072

[B24] Khakdan F. Alizadeh H. Ranjbar M. (2018). Molecular cloning, functional characterization and expression of a drought inducible phenylalanine ammonia-lyase gene (ObPAL) from Ocimum basilicum L. Plant Physiol. Biochem. 130, 464–472. doi: 10.1016/j.plaphy.2018.07.026 30077922

[B25] Kulikov S. N. Chirkov S. N. Il'ina A. V. Lopatin S. A. Varlamov V. P. (2006). Effect of the molecular weight of chitosan on its antiviral activity in plants. Prikl. Biokhim Mikrobiol. 42, 224–228. doi: 10.1134/s0003683806020165 16761579

[B26] Li C. Dong S. Liu X. Guan J. Beckles D. M. Gu X. . (2025a). Co-domestication of cold tolerance and female flower is determined by CsEIN2 in cucumber. Plant Biotechnol. J. 23, 4412–4427. doi: 10.1111/pbi.70195 40622842 PMC12483969

[B27] Li B. Liu B. Shan C. Ibrahim M. Lou Y. Wang Y. . (2013a). Antibacterial activity of two chitosan solutions and their effect on rice bacterial leaf blight and leaf streak. Pest Manag Sci. 69, 312–320. doi: 10.1002/ps.3399 23129534

[B28] Li K. Liu S. Xing R. Qin Y. Li P. (2013b). Preparation, characterization and antioxidant activity of two partially N-acetylated chitotrioses. Carbohydr. Polym. 92, 1730–1736. doi: 10.1016/j.carbpol.2012.11.028 23399213

[B29] Li H. Yang X. Mao M. Xue X. Yao G. Zhang Q. . (2025b). 24-Epibrassinolide treatment alleviates frost damage of apple flower via regulating proline, ROS, and energy metabolism. Plant Physiol. Biochem. 220, 109507. doi: 10.1016/j.plaphy.2025.109507 39864298

[B30] Li Y. Zhang Q. Ou L. Ji D. Liu T. Lan R. . (2020). Response to the cold stress signaling of the tea plant (Camellia sinensis) elicited by Chitosan oligosaccharide. Agronomy 10, 915. doi: 10.3390/agronomy10060915 30654563

[B31] Liaqat F. Eltem R. (2018). Chitooligosaccharides and their biological activities: A comprehensive review. Carbohydr. Polym. 184, 243–259. doi: 10.1016/j.carbpol.2017.12.067 29352917

[B32] Limpanavech P. Chaiyasuta S. Vongpromek R. Pichyangkura R. Khunwasi C. Chadchawan S. . (2008). Chitosan effects on floral production, gene expression, and anatomical changes in the Dendrobium orchid. Sci. Hortic. 116, 65–72. doi: 10.1016/j.scienta.2007.10.034 38826717

[B33] Liu X. Wang S. Zeng K. Li W. Wang S. Huang S. . (2025). N-myristoyltransferase1 regulates biomass accumulation in cucumber (Cucumis sativus L.). J. Integr. Agric. 24, 1754–1768. doi: 10.1016/j.jia.2024.01.013 38826717

[B34] Lu B.-Y. Cheng G.-X. Zhang Z. Sun J.-T. Ali M. Jia Q.-L. . (2019). CaMYC, A novel transcription factor, regulates anthocyanin biosynthesis in color-leaved pepper (Capsicum annuum L.). J. Plant Growth Regul. 38, 574–585. doi: 10.1007/s00344-018-9871-2 30311153

[B35] Lv Y. Yang M. Hu D. Yang Z. Ma S. Li X. . (2017). The OsMYB30 transcription factor suppresses cold tolerance by interacting with a JAZ protein and suppressing β-amylase expression. Plant Physiol. 173, 1475–1491. doi: 10.1104/pp.16.01725 28062835 PMC5291022

[B36] MacDonald M. J. D'Cunha G. B. (2007). A modern view of phenylalanine ammonia lyase. Biochem. Cell Biol. 85, 273–282. doi: 10.1139/o07-018 17612622

[B37] Mao D. Xin Y. Tan Y. Hu X. Bai J. Liu Z. . (2019). Natural variation in the HAN1 gene confers chilling tolerance in rice and allowed adaptation to a temperate climate. Proc. Natl. Acad. Sci. 116, 3494–3501. doi: 10.1073/pnas.1819769116 30808744 PMC6397538

[B38] Meyers K. J. Watkins C. B. Pritts M. P. Liu R. H. (2003). Antioxidant and antiproliferative activities of strawberries. J. Agric. Food. Chem. 51, 6887–6892. doi: 10.1021/jf034506n 14582991

[B39] Naing A. H. Kim C. K. (2021). Abiotic stress-induced anthocyanins in plants: Their role in tolerance to abiotic stresses. Physiol. Plant 172, 1711–1723. doi: 10.1111/ppl.13373 33605458

[B40] Peng X. Wu H. Chen H. Zhang Y. Qiu D. Zhang Z. (2019). Transcriptome profiling reveals candidate flavonol-related genes of Tetrastigma hemsleyanum under cold stress. BMC Genomics 20, 687. doi: 10.1186/s12864-019-6045-y 31472675 PMC6717372

[B41] Pérez-Pérez R. Pinski A. Zaranek M. Beckmann M. Mur L. A. J. Nowak K. . (2025). Effect of potent inhibitors of phenylalanine ammonia-lyase and PVP on *in vitro* morphogenesis of Fagopyrum tataricum. BMC Plant Biol. 25, 469. doi: 10.1186/s12870-025-06440-x 40229725 PMC11998252

[B42] Petridis A. Döll S. Nichelmann L. Bilger W. Mock H. P. (2016). Arabidopsis thaliana G2-LIKE FLAVONOID REGULATOR and BRASSINOSTEROID ENHANCED EXPRESSION1 are low-temperature regulators of flavonoid accumulation. New Phytol. 211, 912–925. doi: 10.1111/nph.13986 27125220

[B43] Pratyusha D. S. Sarada D. V. L. (2022). MYB transcription factors—master regulators of phenylpropanoid biosynthesis and diverse developmental and stress responses. Plant Cell Rep. 41, 2245–2260. doi: 10.1007/s00299-022-02927-1 36171500

[B44] Qin Y. Li Q. An Q. Li D. Huang S. Zhao Y. . (2022). A phenylalanine ammonia lyase from Fritillaria unibracteata promotes drought tolerance by regulating lignin biosynthesis and SA signaling pathway. Int. J. Biol. Macromol. 213, 574–588. doi: 10.1016/j.ijbiomac.2022.05.161 35643154

[B45] Quan J. Zheng W. Tan J. Li Z. Wu M. Hong S. B. . (2022). Glutamic Acid and Poly-γ-glutamic Acid Enhanced the Heat Resistance of Chinese Cabbage (Brassica rapa L. ssp. pekinensis) by Improving Carotenoid Biosynthesis, Photosynthesis, and ROS Signaling. Int. J. Mol. Sci. 23 (19), 11671. doi: 10.3390/ijms231911671 36232971 PMC9570168

[B46] Shang Q.-M. Li L. Dong C.-J. (2012). Multiple tandem duplication of the phenylalanine ammonia-lyase genes in Cucumis sativus L. Planta 236, 1093–1105. doi: 10.1007/s00425-012-1659-1 22572777

[B47] Tan C. Li N. Wang Y. Yu X. Yang L. Cao R. . (2023). Integrated physiological and transcriptomic analyses revealed improved cold tolerance in cucumber (Cucumis sativus L.) by exogenous Chitosan oligosaccharide. Int. J. Mol. Sci. 24 (7), 6202. doi: 10.3390/ijms24076202 37047175 PMC10094205

[B48] Tan K. Song X. Xu Z. Zhu H. Zhang Y. Xu S. . (2026). The MdICE1/MdFAMA-MdTYDC transcriptional module confers cold tolerance by regulating dopamine metabolism in apple. Plant Biotechnol. J. 24 (5), 3012–3031. doi: 10.1111/pbi.70544 41540774 PMC13110186

[B49] Vannini C. Locatelli F. Bracale M. Magnani E. Marsoni M. Osnato M. . (2004). Overexpression of the rice Osmyb4 gene increases chilling and freezing tolerance of Arabidopsis thaliana plants. Plant J. 37, 115–127. doi: 10.1046/j.1365-313x.2003.01938.x 14675437

[B50] Xing J. Ye X. Huo K. Ding Z. Tie W. Xie Z. . (2025). Integrated metabolomic and transcriptomic analyses revealed the overlapping response mechanisms of banana to cold and drought stress. Plant Physiol. Biochem. 222, 109766. doi: 10.1016/j.plaphy.2025.109766 40086128

[B51] Yan Y. Mintao S. Si M. Qian F. Yijia W. Qinghua D. . (2022). Mechanism of CsGPA1 in regulating cold tolerance of cucumber. Hortic. Res. 9, uhac109. doi: 10.1093/hr/uhac109 35821703 PMC9265480

[B52] Yang A. Dai X. Zhang W. H. (2012). A R2R3-type MYB gene, OsMYB2, is involved in salt, cold, and dehydration tolerance in rice. J. Exp. Bot. 63, 2541–2556. doi: 10.1093/jxb/err431 22301384 PMC3346221

[B53] Yang X. Luo Y. Bai H. Li X. Tang S. Liao X. . (2022). DgMYB2 improves cold resistance in chrysanthemum by directly targeting DgGPX1. Hortic. Res. 9, uhab028. doi: 10.1093/hr/uhab028 35039835 PMC8801720

[B54] Zeng R. Shi Y. Guo L. Fu D. Li M. Zhang X. . (2025). A natural variant of COOL1 gene enhances cold tolerance for high-latitude adaptation in maize. Cell 188, 1315–1329.e1313. doi: 10.1016/j.cell.2024.12.018 39842436

[B55] Zhang Q. Chen Q. Wang S. Hong Y. Wang Z. (2014). Rice and cold stress: methods for its evaluation and summary of cold tolerance-related quantitative trait loci. Rice 7, 24. doi: 10.1186/s12284-014-0024-3 25279026 PMC4182278

[B56] Zhang Y. Fan L. Zhao M. Chen Q. Qin Z. Feng Z. . (2019). Chitooligosaccharide plays essential roles in regulating proline metabolism and cold stress tolerance in rice seedlings. Acta Physiol. Plant 41, 77. doi: 10.1007/s11738-019-2864-3 30311153

[B57] Zhang H. Li T. Ma X. Zhao P. Gao X. Sun Y. . (2026). Cordyceps militaris polysaccharides improved tomato postharvest disease resistance. Postharvest Biol. Technol. 231, 113881. doi: 10.1016/j.postharvbio.2025.113881 38826717

[B58] Zhao Z. Li J. Zhao X. Wu S. Zhang Y. Liu Z. . (2025a). Mutation of a magnesium chelatase BrCHLD affects the chlorophyll content and magnesium chelatase activity in Chinese cabbage. Plant Physiol. Biochem. 228, 110319. doi: 10.1016/j.plaphy.2025.110319 40749464

[B59] Zhao Z. Pan F. Zhao T. Zhang L. Hou Q. Tang T. . (2025b). A single nucleotide mutation in BrECB2 impaired RNA editing efficiency and early chloroplast biosynthesis. J. Integr. Agric. doi: 10.1016/j.jia.2025.11.001 38826717

[B60] Zhao Z. Tan C. Zhang J. Zhang L. Hou Q. Tang T. . (2025c). BrSWN mutation reduces the H3K27me3 level at the BrFLC2 and BrFLC3 loci and confers a late-bolting phenotype in Chinese cabbage. Plant J. 122, e70151. doi: 10.1111/tpj.70151 40226975

[B61] Zhao Z. Zhang L. Zhang M. Tan C. Zhang Y. Liu Z. . (2026). A 5-bp deletion in BrGL1 leads to glabrous leaves in Chinese cabbage. Physiol. Plant 178, e70752. doi: 10.1111/ppl.70752 41574391

[B62] Zhao Z. Zhao X. Wu S. Zhang Y. Feng H. Liu Z. . (2025d). A single-base mutation on the 5' UTR of BrATG5 confers the premature leaf senescence phenotype in Chinese cabbage. Theor. Appl. Genet. 138, 235. doi: 10.1007/s00122-025-05026-3 40885882

[B63] Zhishen J. Mengcheng T. Jianming W. (1999). The determination of flavonoid contents in mulberry and their scavenging effects on superoxide radicals. Food Chem. 64, 555–559. doi: 10.1016/S0308-8146(98)00102-2

[B64] Zhou J. Chen Q. Zhang Y. Fan L. Qin Z. Chen Q. . (2018). Chitooligosaccharides enhance cold tolerance by repairing photodamaged PS II in rice. J. Agric. Sci. 156, 888–899. doi: 10.1017/S0021859618000862 41292463

[B65] Zou P. Tian X. Dong B. Zhang C. (2017). Size effects of chitooligomers with certain degrees of polymerization on the chilling tolerance of wheat seedlings. Carbohydr. Polym. 160, 194–202. doi: 10.1016/j.carbpol.2016.12.058 28115094

