# Supplementary material for: The CsPHL11-CsPAL2 module mediates chitooligosaccharide-induced cold tolerance in cucumber seedlings
Source: Front Plant Sci. 2026 Jun 12;17:1862603. doi: 10.3389/fpls.2026.1862603 (PMC13303117; doi:10.3389/fpls.2026.1862603)
Supplement: Supplementary file 1 [file Table1.docx]

Supplementary Material

**Supplementary Table 1.** Primer used in this study.

| **Primer** | **Sequecne** | **Purpose** |
| --- | --- | --- |
| CsaV3_6G039710-F | TGGATTGGCTTCTATCGTTCTCT | Gene expression |
| CsaV3_6G039710-R | AGGATGATGCTTCAACTTATGGG |  |
| CsaV3_4G026530-F | GCCAGATTCAAACACGCGCT |  |
| CsaV3_4G026530-R | ACTTCACGGAGGCTTCCAACG |  |
| CsaV3_3G008170-F | ATGGGGCAATAGGTGGTCAAA |  |
| CsaV3_3G008170-R | ATCCAAAAGCGGTTGATAATGTC |  |
| CsaV3_4G007760-F | GAACCGAAATCTACTTGGA |  |
| CsaV3_4G007760-R | GGCTCACCACCATACCTC |  |
| CsaV3_6G039670-F | AATCTGCTAGAGCTGGTG |  |
| CsaV3_6G039670-R | CCATAACTATCGGTCCCT |  |
| CsaV3_4G002300-F | TCGGGAGATTTGGTTCCTTTGT |  |
| CsaV3_4G002300-R | ACTCAGAACCGACGGCAGTG |  |
| CsaV3_4G002310-F | TTCGGAGATTTGGTTCCTTTGT |  |
| CsaV3_4G002310-R | GAATCAATACCAGCCTGTGTAAAAG |  |
| CsaV3_2G010620-F | AATCATCTTGGTGGAGCA |  |
| CsaV3_2G010620-R | AGTTGAGCATTGGTTTCG |  |
| CsaV3_3G010950-F | AGGTCTCACCCTTTATCACTT |  |
| CsaV3_3G010950-R | AGATCCTGTGCCATTATTCT |  |
| CsaV3_3G030370-F | TTTTGTAGACGCGGTCAC |  |
| CsaV3_3G030370-R | ATGGCTCTTCAAGTGATA |  |
| CsaV3_2G024920-F | GCTGCGGCAGAATCCAAT |  |
| CsaV3_2G024920-R | GGTCGTCCAAATCATTTATC |  |
| CsaV3_4G002290-F | GCTACCTACCCTCTTATGC |  |
| CsaV3_4G002290-R | CGTTCGTTTCGCTATCAC |  |
| CsaV3_4G002320-F | AACTGGCGAAAGGGTAAT |  |
| CsaV3_4G002320-R | GAATTGGTGTTCCATCCC |  |
| CsaV3_4G002330-F | AAGTTGTTGACAGGGAATA |  |
| CsaV3_4G002330-R | TGCATAAGTGGGTAGGTG |  |
| CsaV3_6G015190-F | TCTCCCTGGTCTTGCTGT |  |
| CsaV3_6G015190-R | GAAGGCTTTCATCCTTGC |  |
| CsaV3_6G039680-F | GCTGAAGGCTGTTCTACCC |  |
| CsaV3_6G039680-R | GCAAATTAGTGCCCAACG |  |
| CsaV3_6G039690-F | TTGTGCATTGGCTCTAAC |  |
| CsaV3_6G039690-R | CTCCGATACTCCTCAACC |  |
| CsaV3_6G039720-F | GTGAGGACTAACACCCTA |  |
| CsaV3_6G039720-R | AGTAACAGTTCCACGAAG |  |
| CsPAL2-F | CTGCAGGGGCCCGGGGTCGAC ATGGCACCAATTGAGACCACA | Subcellular localization |
| CsPAL2-R | GCCCTTGCTCACCATGGTACC ACATATGGGAATGGGAGCTCC |  |
| CsPHL11-AD-F | GCCATGGAGGCCAGTGAATTC ATGGAGCGGAATTACCCCTAT | Y1H |
| CsPHL11-AD-R | AGCTCGAGCTCGATGGATCC CCTTGAACAAGGAAGCACTTT |  |
| ProCsPAL2-pAbAi-F | GAAAAGCTTGAATTCGAGCTC GATTTAATAACATATTCTGAATAAATTTATCCTT |  |
| ProCsPAL2-pAbAi-R | ATACAGAGCACATGCCTCGAG TTTGTGATTTAGCTACTTGTATATTAGTGGT |  |
| CsPHL11-F | GAGCTCGGTACCCGGGGATCC ATGGAGCGGAATTACCCCTAT | Dual-LUC |
| CsPHL11-R | GCCCTTGCTCACCATGTCGAC CCTTGAACAAGGAAGCACTTT |  |
| ProCsPAL2-LUC-F | GCCCCCCCTCGAGGTCGAC GATTTAATAACATATTCTGAATAAATTTATCCTT |  |
| ProCsPAL2-LUC-R | CTCTAGAACTAGTGGATCC TTTGTGATTTAGCTACTTGTATATTAGTGGT |  |
| CsPAL2-F | GTGAGTAAGGTTACCGAATTC TATTTTGAAACTCATGTCTTCCACCT | VIGS |
| CsPAL2-R | CGTGAGCTCGGTACCGGATCC TCGCCATTTGCAAGTGCG |  |
| CsPHL11-F | GTGAGTAAGGTTACCGAATTC TGGAGCGGAATTACCCCTAT |  |
| CsPHL11-R | CGTGAGCTCGGTACCGGATCC TCTACTCCGGAGAAATTGCTAA |  |
